# A Review of Trend of Nursing Theories related Caregivers in Korea

**DOI:** 10.2174/1874434601812010045

**Published:** 2018-03-30

**Authors:** Sung Hae Kim, Yoona Choi, Ji-Hye Lee, Da-El Jang, Sanghee Kim

**Affiliations:** 1 College of Nursing, Graduate School, Yonsei University, Seoul, Korea; 2 College of Nursing, Graduate School, Yonsei University, Samsung Medical Center, Seoul, Korea; 3 College of Nursing, Graduate School, Yonsei University, Severance hospital, Seoul, Korea; 4 College of Nursing, Mo-Im Kim Nursing Research Institute, Yonsei University, Seoul, Korea

## A Review of Trend of Nursing Theories related Caregivers in Korea. The Open Nursing Journal: 12-26.

The correct names are provided and replaced online which is mentioned as under:

Sung Hae Kim^1^, Yoona Choi^1^,*, Ji-Hye Lee^2^, Da-El Jang^3^ and Sanghee Kim^4^

^1^College of Nursing, Graduate School, Yonsei University, Seoul, Korea

^2^College of Nursing, Graduate School, Yonsei University, Samsung Medical Center, Seoul, Korea

^3^College of Nursing, Graduate School, Yonsei University, Severance hospital, Seoul, Korea

^4^College of Nursing, Mo-Im Kim Nursing Research Institute, Yonsei University, Seoul, Korea

The original names was: 

Sung Hae Kim^1^, Yoona Choil^1*^, Ji-Hye Lee^2^, Da-El Jang^3^, Sanghee Kim^1^

